# Rapid Transient Production in Plants by Replicating and Non-Replicating Vectors Yields High Quality Functional Anti-HIV Antibody

**DOI:** 10.1371/journal.pone.0013976

**Published:** 2010-11-12

**Authors:** Frank Sainsbury, Markus Sack, Johannes Stadlmann, Heribert Quendler, Rainer Fischer, George P. Lomonossoff

**Affiliations:** 1 Department of Biological Chemistry, John Innes Centre, Norwich, United Kingdom; 2 Institute for Molecular Biotechnology, Rheinisch-Westfälische Technische Hochschule (RWTH), Aachen, Germany; 3 Department for Chemistry, University of Natural Resources and Applied Life Sciences, Vienna, Austria; 4 Institute of Applied Microbiology, University of Natural Resources and Applied Life Sciences, Vienna, Austria; 5 Fraunhofer Institute for Molecular Biology and Applied Ecology, Aachen, Germany; University of California San Francisco, United States of America

## Abstract

**Background:**

The capacity of plants and plant cells to produce large amounts of recombinant protein has been well established. Due to advantages in terms of speed and yield, attention has recently turned towards the use of transient expression systems, including viral vectors, to produce proteins of pharmaceutical interest in plants. However, the effects of such high level expression from viral vectors and concomitant effects on host cells may affect the quality of the recombinant product.

**Methodology/Principal Findings:**

To assess the quality of antibodies transiently expressed to high levels in plants, we have expressed and characterised the human anti-HIV monoclonal antibody, 2G12, using both replicating and non-replicating systems based on deleted versions of *Cowpea mosaic virus* (CPMV) RNA-2. The highest yield (approximately 100 mg/kg wet weight leaf tissue) of affinity purified 2G12 was obtained when the non-replicating CPMV-*HT* system was used and the antibody was retained in the endoplasmic reticulum (ER). Glycan analysis by mass-spectrometry showed that the glycosylation pattern was determined exclusively by whether the antibody was retained in the ER and did not depend on whether a replicating or non-replicating system was used. Characterisation of the binding and neutralisation properties of all the purified 2G12 variants from plants showed that these were generally similar to those of the Chinese hamster ovary (CHO) cell-produced 2G12.

**Conclusions:**

Overall, the results demonstrate that replicating and non-replicating CPMV-based vectors are able to direct the production of a recombinant IgG similar in activity to the CHO-produced control. Thus, a complex recombinant protein was produced with no apparent effect on its biochemical properties using either high-level expression or viral replication. The speed with which a recombinant pharmaceutical with excellent biochemical characteristics can be produced transiently in plants makes CPMV-based expression vectors an attractive option for biopharmaceutical development and production.

## Introduction

Plant viruses have been used as vectors for the expression of recombinant proteins for over 20 years. Recently, a number of pharmaceutically relevant proteins have been produced using vectors based on full-length plant virus genomes [1], [2]. Though such vectors have the advantage that they can spread systemically within a plant and can be readily transmitted in order to bulk up material, they suffer from disadvantages in terms of the size of insert which can be stably incorporated and raise issues of biocontainment. As a result, attention has turned towards the development of deconstructed or deleted versions of plant virus-based expression systems that can alleviate the disadvantages of full-length viral vectors while retaining speed and high productivity. Deleted versions of the RNA viruses, *Tobacco mosaic virus* (TMV), *Potato virus X* (PVX), and *Cowpea mosaic virus* (CPMV) RNA-2 have successfully been used been used to produce a variety of proteins in plants [3]–[6]. In these vectors the region encoding the coat protein(s) was removed, limiting the ability of the virus to spread within the plant but providing a substantial measure of biocontainment. High level expression is achieved by retaining the ability of the viral RNA to be replicated by its cognate RNA-dependent RNA polymerase and through the efficient delivery of the constructs to cells by agro-infiltration.

A potential drawback of replicating virus-based expression systems, which has to date received little attention, is that expression of viral proteins [7], as well as the regulation of host proteome associated with viral replication [8], [9], causes substantial changes to the host cells. For example, expression of the replication-related proteins encoded by CPMV RNA-1 is known to induce a massive proliferation of endoplasmic reticulum (ER)-derived membranes [10]–[12]. Since the ER is essential for folding and post-translational modification of glycoproteins such as antibodies, perturbations to the endomembrane system could result in a reduction in quality of recombinant protein or in different post-translational modification patterns (including N-glycosylation). On the other hand, an increase in ER-derived membranes, as observed in differentiated plasma B-cells, can have a beneficial effect by increasing capacity for the accumulation of immunoglobulins. Furthermore, the high levels of protein synthesis which can be achieved using viral vectors could potentially affect the quality of the protein by, for example, saturating certain host components necessary for quality control or post-translational modification.

The properties of a recombinant pharmaceutical, such as an antibody, are clearly crucial for its proper function and have, therefore, been studied extensively in a number of production systems. Recently, the broadly neutralising anti-*Human immunodeficiency virus* (HIV) human monoclonal antibody (mAb), 2G12 [13], [14], has attracted considerable interest as a microbicide for preventing the spread of HIV. This antibody recognizes a highly conserved epitope consisting of high-mannose N-glycans on the HIV-1 gp120 envelope protein [14] and has a potent and broad HIV-1-neutralizing activity *in vitro* and *in vivo* [15]. Studies in primates have demonstrated the ability of 2G12 to control infection and prevent transmission when supplied parenterally and through mucosal tissue [16]–[19]. Antibody cocktails including 2G12 have proven able to reduce the rate of viral rebound after ending antiretroviral treatment in some human patients [20], but the use of these cocktails requires multiple high doses. To assess whether plants could serve as a source of large quantities of 2G12, the full-size antibody has been expressed in transgenic Arabidopsis, tobacco and maize [21]–[23]. These reports rigorously and indisputably demonstrated the potential and flexibility of recombinant antibody production afforded by expression in plants. However, the use of transient expression systems, both viral and non-viral, can deliver significant advantages for recombinant protein production over transgenic plants in terms of yield and speed of expression.

We have previously shown that replication-competent versions of a deleted version of CPMV RNA-2 (delRNA-2)[24] can be used to produce assembled IgG molecules in plants [25]. While the delRNA-2 system relies on the simultaneous presence RNA-1 for its replication, we have shown that very high levels of assembled 2G12 molecules can be produced using the non-replicating CPMV-*HT* system, which does not require the presence of RNA-1 [26]. The present study reports the results of an investigation into the biochemical properties and *in vitro* activity and neutralisation capabilities of purified 2G12 produced by transient expression in *Nicotiana benthamiana* using these replicating and non-replicating systems. In particular, the effect of the ER remodelling caused by RNA-1 on the quality of purified antibody was assessed. The results show that neither the presence of RNA-1 nor high levels of transient expression achieved with the CPMV-*HT* system have marked effects on the properties and efficacy of 2G12 when compared to the Chinese hamster ovary (CHO) cell-produced antibody. Thus, in view of the speed advantage over transgenic plants, CPMV-based transient expression vectors offer an attractive option for development of plant-produced pharmaceuticals.

## Results

### Purification of ^CPMV^2G12 produced in plants by delRNA-2 plus RNA-1 or CPMV-*HT*

**Figure 1:**
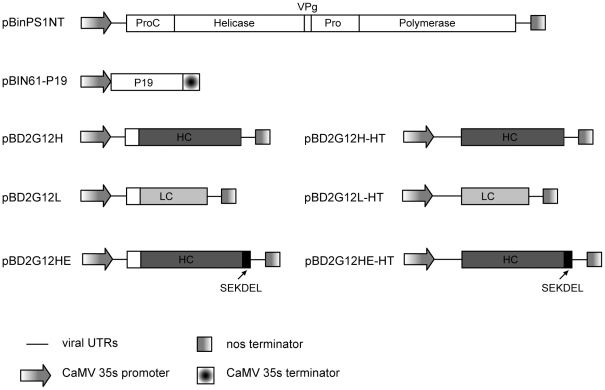
Schematic representation of the plant expression cassettes used to express 2G12. White boxes represent CPMV coding sequences, light and dark grey boxes represent 2G12 light and heavy chains respectively.

To examine the properties of 2G12 transiently expressed in *N. benthamiana* using both replicating and non-replicating CPMV vectors, two series of plasmids were used. These consisted of the Heavy (H) chain, with and without a KDEL ER-retention sequence at its C-terminus or Light (L) chain, inserted into either delRNA-2 or CPMV-*HT* (Figure 1). *Agrobacterium* suspensions containing the various versions of the H chain were mixed with the L chain and with equal amounts of an *Agrobacterium* suspension containing plasmid pBIN61-P19, which expresses the suppressor of silencing P19. For the delRNA-2 constructs an *Agrobacterium* suspension containing plasmid pBinPS1NT, which is a full-length copy of CPMV RNA-1, was also added. The mixtures were used to agroinfiltrate all fully-expanded leaves of 6-week old *N. benthamiana*, from which tissue was harvested at 6 days post-infiltration and the IgG molecules purified by protein-A affinity chromatography. At each step of the purification, the concentration of 2G12 was monitored by surface-plasmon resonance (SPR; Table 1). The yields of IgG produced using CPMV-*HT* (*^HT^*2G12HL and *^HT^*2G12HEL) were significantly higher (approximately 5-fold) than when delRNA-2 was used in the presence of RNA-1 (^CPMV^2G12HL and ^CPMV^2G12HEL). With both systems, higher levels of antibody were obtained when KDEL sequence was present.

**Table 1:** Purification of ^CPMV^2G12 and *^HT^*2G12 variants from infiltrated tissue.

2G12 variant	Leaf mass (g)	Extract volume (ml)	2G12 conc. (µg/ml)	2G12 yield (mg)	% Recovery	mg recovered/kg of fresh weight tissue
^CPMV^2G12HL	176.0	485	5.37	2.6	73%	10.8
*^HT^*2G12HL	134.9	435	20.57	9.0	79%	52.6
^CPMV^2G12HEL	105.9	315	12.75	4.0	71%	26.9
*^HT^*2G12HEL	85.6	290	36.56	10.6	85%	105.1

### Integrity of ^CPMV^2G12 and *^HT^*2G12

**Figure 2:**
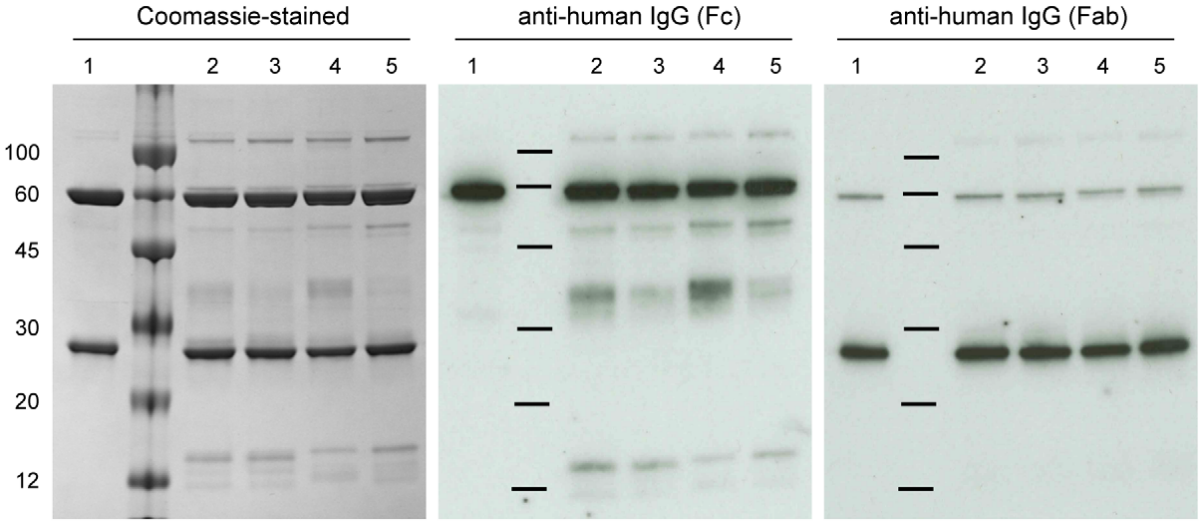
Electrophoretic analysis of protein-A purified 2G12 variants produced by delRNA-2 + RNA-1 or with CPMV-*HT*. 12% SDS-PAGE under reducing conditions was used to separate 4 µg of 2G12 preparations for Coomassie staining and 50 ng for immunological detection of samples from ^CHO^2G12 (1), ^CPMV^2G12HL (2), *^HT^*2G12HL (3), ^CPMV^2G12HEL (4), and *^HT^*2G12HEL (5). A sample of size markers (visible on the Coomassie-stained gel but not the blots) was loaded between lanes 1 and 2.

The integrity of the purified 2G12 was assessed by SDS-PAGE and Western blot analysis under reducing conditions with CHO-produced 2G12 providing a protein standard (Figure 2). The Coomassie blue-stained gel showed that the main products were the H and L chains which co-migrated with the equivalent bands from CHO-produced 2G12 (^CHO^2G12). The plant-produced material contained some additional protein bands, not present in the CHO-produced material, which Western blot analysis using anti-Fc and anti-Fab antibody identified as H chain-derived degradation products. Furthermore, a doublet of ∼110 kDa is present in the material purified from plants, which probably corresponds to cross-linked H chain dimers. Thus, the plant derived 2G12 is not as pure as the ^CHO^2G12 due to the presence of some product-related impurities. The presence of degradation products after a single-step protein-A affinity purification has been previously observed [22], [27], [28], and these contaminants can be removed by additional purification steps. Importantly, this analysis showed no significant differences between 2G12 produced by delRNA-1 with RNA-1 or by CPMV-*HT*.

### Glycan analysis of ^CPMV^2G12 and *^HT^*2G12

**Figure 3:**
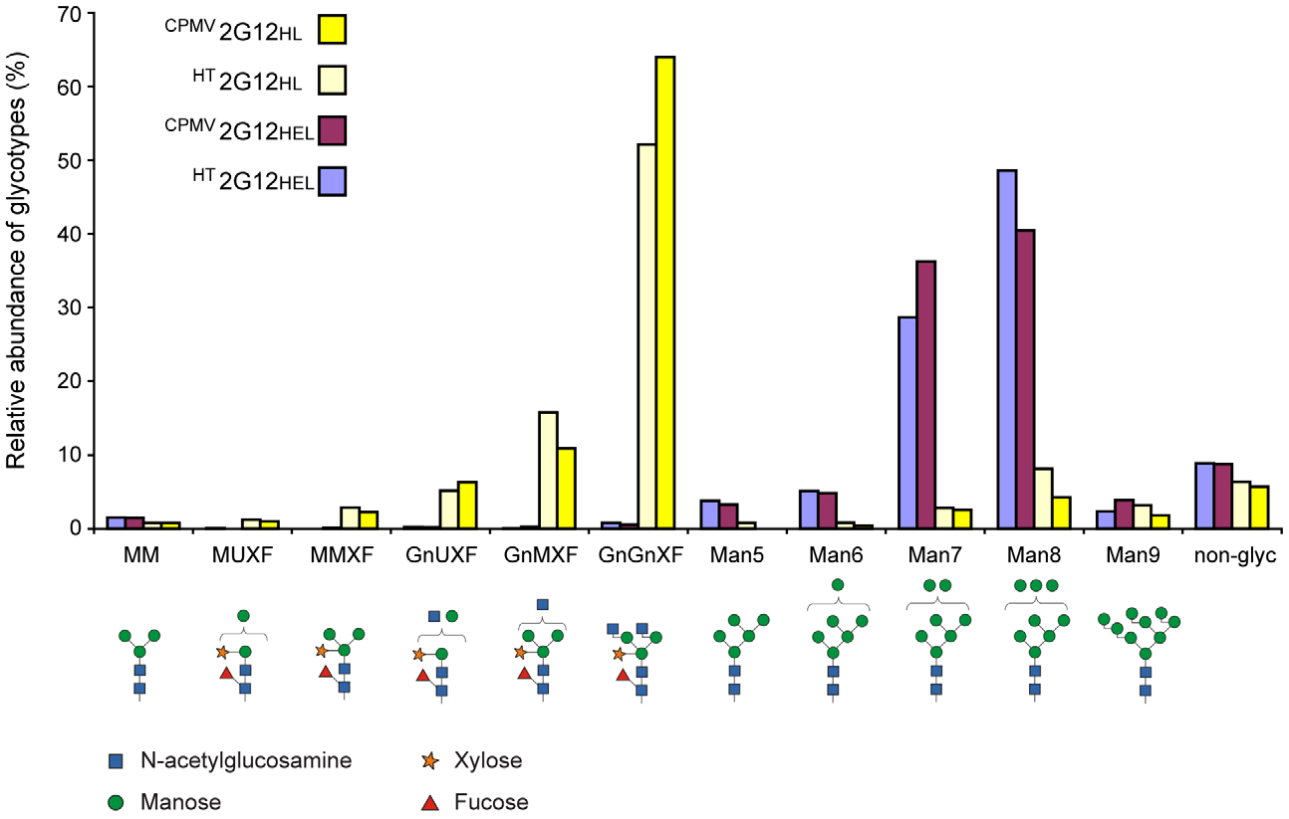
Relative abundances of glycoforms for each of the purified 2G12 variants with diagrammatic structures of each glycotype. Data was generated by mass-spectrometric analysis of the two predicted tryptic peptides representing the same glycosylation site (EEQYN^297^STYR and TKPREEQYN^297^STYR). *N*-glycan structure abbreviations are given according to http://www.proglycan.com.

The glycosylation of the plant-produced antibody preparations was of particular interest due to the potential of the remodelling of the endomembrane system by RNA-1 to affect protein maturation and modification. Mass spectrometry was used to analyse the N-linked glycan profiles of purified samples of both ER-retained (2G12HEL) and secreted (2G12HL) forms expressed from delRNA-2 in the presence of RNA-1 or by CPMV-*HT* in its absence. As expected the ER-retained forms, ^CPMV^2G12HEL and *^HT^*2G12HEL, displayed mostly oligo-mannosidic type glycans, with Man-7 and Man-8 predominating, typical of KDEL-tagged antibodies. Only residual amounts of complex type N-glycans were found (total <2%), showing that KDEL-mediated ER retrieval of 2G12 is highly efficient. In contrast, the majority of glycan structures on ^CPMV^2G12HL and *^HT^*2G12HL were plant-specific complex type residues typical of secretion in plant cells (Figure 3). The major N-glycans GnGnXF and GnMXF accounted for 74.9% for ^CPMV^2G12HL and 67.8% for *^HT^*2G12HL. Only a small fraction of oligo-mannosidic type N-glycans (total <16%) were found, showing that exit from the ER was efficient for both ^CPMV^2G12HL and *^HT^*2G12HL.

The observed glycan profiles for 2G12 expressed by both delRNA-2 in the presence of RNA-1 and by CPMV-*HT* do not differ significantly from each other. Furthermore, whether ER-retained or secreted, the glycosylation patterns are similar to those from 2G12 expressed in other systems [27](Sack M and Stadlmann J, unpublished observations) and to other antibodies produced in plants [29]. In the case of delRNA-2 with RNA-1, further investigations showed that an in-plant incubation time of 12 days, as opposed to the usual 6 days, revealed a positive effect of the presence of RNA-1 on accumulation levels of ER-retained 2G12. Although there was a shift in the predominance of a particular glycotype over time, only minor differences in the N-linked glycan profile were observed for 2G12 samples at 6 or 12 days between the presence and absence of RNA-1 (Figure S1). Therefore, neither the presence of RNA-1 nor the high-level accumulation from CPMV-*HT* expression impacted greatly on 2G12 glycosylation.

### Quality and activity analysis of ^CPMV^2G12 and *^HT^*2G12 by Surface Plasmon Resonance

**Figure 4:**
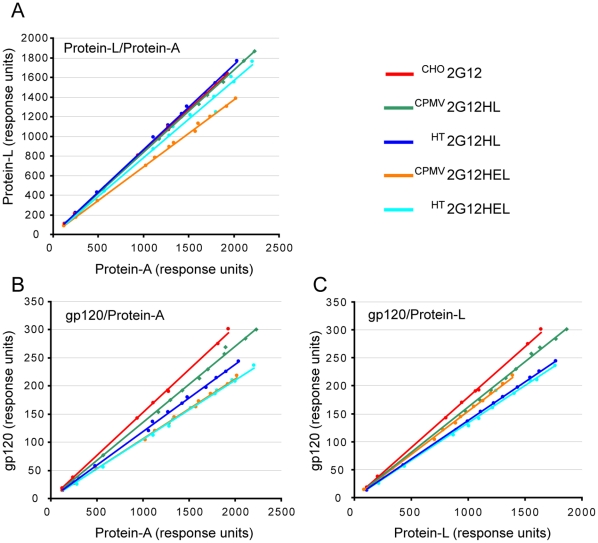
Ratios of the binding rate signals obtained by SPR on the purified 2G12 variants. Values were derived using protein-A, protein-L, and gp120 surfaces represented by linear regressions. (A) protein-L/protein-A ratio. (B) gp120/protein-A ratio. (C) gp120/protein-L ratio.

The binding properties of purified delRNA-2 and CPMV-*HT*-produced 2G12 were evaluated by SPR-based binding assays using surfaces immobilized with protein-A, protein-L or HIV gp120. Each immobilized ligand probes a specific binding region on the antibody and the ratios of the measured response can be used to compare and evaluate the quality of antibody preparations. Inactivation of a ligand binding site can result from absence of an antibody chain, inappropriate assembly, misfolding, or unexpected modification of the binding site, and will affect the response ratios. The response ratios were directly derived by linear regression of the endpoint signals measured for different concentrations (Figure 4) and the relative response ratios are reported in Table 2. The ^CHO^2G12 was again employed as a reference standard. For the plant-produced material, the gp120/protein-A ratio varied from 69 to 89% of the response ration of ^CHO^2G12 and the gp120/protein-L ratio was 74 to 90% of the response ration of ^CHO^2G12. These results show that the plant-produced material is slightly inferior to the more highly purified CHO-produced material. The observed differences can only partially be explained by the presence of H chain degradation products (Figure 2) because the gp120/protein-L ratios are also slightly reduced. The reduction in this ratio is somewhat more prominent for CPMV-*HT*-produced 2G12 and may indicate incomplete or improper assembly of the antibody that could be due to the disruption of host cell processes by the high-level over-expression of a foreign protein. However, it may also be related to the extraction and purification of different batches of antibody, for which there is inherent variation in any production system.

**Table 2:** Response Ratios determined for ^CPMV^2G12 and *^HT^*2G12 variants relative to ^CHO^2G12.

2G12 variant	Protein-L/Protein-A	gp120/Protein-A	gp120/Protein-L
^CPMV^2G12HL	98.82	88.80	89.84
*^HT^*2G12HL	101.97	78.19	76.62
^CPMV^2G12HEL	81.09	69.61	85.84
*^HT^*2G12HEL	92.44	68.96	74.46

### HIV-1 neutralisation by ^CPMV^2G12 and *^HT^*2G12

The ability of the purified IgG molecules to neutralise HIV was determined via a syncytium inhibition assay using a T cell line-adapted HIV-1 isolate, HIV-1 RF (Table 3). In each case the amount of antibody provided was determined by SPR using a protein-A surface. The results show that all the preparations of purified 2G12, whether produced by delRNA-2 + RNA-1 or CPMV-*HT*, neutralise HIV *in vitro* with the IC_50_ measurements similar to that obtained using CHO-produced 2G12.

**Table 3:** Virus neutralisation by ^CPMV^2G12 and *^HT^*2G12 variants, and ^CHO^2G12.

2G12 variant	^CHO^2G12	^CPMV^2G12HL	*^HT^*2G12HL	^CPMV^2G12HEL	*^HT^*2G12HEL
**IC50 (µg/ml)**	3.72	3.13	4.42	3.41	6.81

## Discussion

The work reported in this manuscript describes an extensive characterisation of a purified full-length IgG produced transiently in plants using both replicating and non-replicating CPMV-based systems. This builds on our previous studies [25], [26] showing that full-length antibodies can be successfully expressed to high levels using such systems. The results show that 2G12 expressed using both systems can be purified in substantial quantities, and retains the ability to bind HIV gp120 and to neutralise HIV infections *in vitro* at levels similar to the CHO-produced standard antibody. Therefore, by comparison to previous studies that have used transgenic plants to produce 2G12, we show that the potential impact of transient high-level expression systems on plant host cell processes, has a negligible effect on recombinant antibody characteristics.

There have been several previous reports of the successful production of full-size antibodies in plants using both replicating and non-replicating transient expression systems. Using two replicating TMV constructs separately expressing the H and L chains of mAb CO17-1A, Verch et al. [30] were able to demonstrate the assembly of full-length antibodies in *N. benthamiana* but did not provide any information as to their yield or properties. Using a non-replicating, non-viral system, Vaquero et al. [31] transiently expressed mAb T84.66 specific for the human carcinoembryonic antigen in *N. tabacum* and achieved a yield of approximately 1 mg purified T84.66 per kg of fresh weight leaf material but no characterisation of the plant-expressed antibody was reported. Giritch et al. [32] and Huang et al. [33] showed that it is possible to produce high-levels of a full-length antibody in *N. Benthamiana*, using non-competing viral vectors based on TMV and PVX, or a single DNA virus-based replicon, respectively. Both studies demonstrated antigen binding and reported the purification of the antibody, but did not give final yields of the purification or conduct a detailed analysis of the product. Activity, in the form of neutralisation assays, was demonstrated for the chimaeric mAb cPIPP, which reached expression levels of 20 mg/kg fresh weight leaf tissue in *N. tabacum* from non-viral transient expression [34]. This chimaeric antibody was later characterised in terms of its glycosylation profile [28]. More recently, Vezina et al. [35] and Villani et al [36] reported the transient expression of antibodies C5-1 and H10, respectively, also without replication, in *N. benthamiana.* In both instances, the purified antibodies were analysed with respect to their glycosylation status and, in the case of H10, the antibody was also subjected to immunohistochemical analysis.

The maximum level of purified 2G12 (105 mg/kg fresh weight tissue) was obtained when the CPMV-*HT* system was used for expression and the antibody was retained in the ER through the incorporation of a KDEL sequence on the H chain. This level is approximately one-third the maximum previously reported as accumulating in infiltrated tissue (325 mg/Kg)[26] when separate constructs are used to express the H and L chains. There are probably two main factors responsible for this reduction: the losses occurring during the purification process, which were 15 to 30% depending on the expression strategy (Table 1), and the nature of the large scale infiltrations. The latter were carried out by hand using syringe-infiltration which is relatively inefficient on a large-scale. Therefore, not all of the tissue included in the extraction process was actually infiltrated, including the mid-vein, which contributes a significant fraction to the weight of the harvested tissue. However, despite this the levels of purified antibodies were similar to that reported by Villani et al. [36] which used vacuum infiltration to deliver Agrobacterium suspensions to *N. benthamiana* tissue. It the absence of ER retention, the yield of antibody dropped by about 50%, consistent with previous observations [26]. When delRNA-2 constructs were used to express 2G12 in the presence of RNA-1, the yields of purified IgG were approximately 5-fold less than when the corresponding constructs were expressed from CPMV-*HT*, which is also consistent with our previous observations and is due to differences in the 5′UTR of CPMV RNA-2 [26].

Glycosylation is an essential post-translational modification for glycoprotein maturation and, in the case of antibodies, may affect activity, immunogenicity, and other secondary functions [37], [38]. Since CPMV RNA-1 is known to induce a proliferation of ER-derived membranes [11], it is possible that this could affect the glycosylation of 2G12 produced using the delRNA-2 system. Likewise, the high levels of antibody produced using CPMV-*HT* might affect glycosylation patterns by overloading the glycosylation machinery. Glycan analysis of the purified 2G12 molecules showed that the pattern of glycosylation depended exclusively on whether the molecule was ER-retained, with no significant differences being found between the delRNA-2 + RNA-1 and the CPMV-*HT*-expressed material. When the 2G12 was retained within the ER, high-mannose forms predominated while in the absence of retention, typical plant-specific glycans were found. Overall, the glycosylation pattern is similar to that found for 2G12 expressed in plant cell culture [27] and in transgenic tobacco plants (Sack M and Stadlmann J, unpublished observations). These observations indicate that neither the membrane proliferation caused by RNA-1, nor the high levels of protein produced in response to CPMV-*HT* affect the post-translational modification of the IgG.

A syncytium inhibition assay demonstrated that 2G12 produced by delRNA-2 + RNA-1 was capable of neutralising HIV *in vitro* at doses very similar to that found with CHO-produced 2G12, irrespective of whether the antibody was ER-retained. The non-ER-retained 2G12 produced from CPMV-*HT* appeared to have a slightly inferior neutralising activity than that of CHO-produced 2G12 while that of the ER-retained version appeared to be worse still. Nonetheless, all the plant-expressed 2G12 preparations described here are still very effective at neutralising HIV and, in fact, their IC_50_s are within the range of IC_50_s reported for CHO- produced 2G12[21], [39]. Importantly, the values are also similar to those of 2G12 produced in transgenic transgenic corn [21], [22] and tobacco (unpublished observations).

When the purified antibodies were subjected to comparison with ^CHO^2G12 in an *in vitro* binding assay, binding response ratios determined by SPR showed that the plant-derived purified antibodies were slightly less active than their CHO-derived counterpart. This can mostly be attributed to the presence of degradation products. The quality of the 2G12 preparations is also slightly lower than that observed for 2G12 derived from transgenic corn [21], [22] and tobacco (unpublished observations). The slight reduction in ligand binding activity observed using the highly accurate and precise assay in this study is not critical for initial biopharmaceutical product development, including biophysical and biochemical characterization and biopotency assays. However, it does suggest that quality issues deserve particular attention in the development of transient expression systems for the production of biopharmaceuticals. The discrepancy in 2G12 quality measurements between stable transgenic and transient expression in plants may be due in part to the physiological state of the leaf material used. Antibody degradation *in planta* can be a function of growth conditions and leaf age [40] and may also be influenced by the defense-related physiological adjustment by the plant that accompanies agro-infiltration [41], which may well include changes to proteolytic activity [42]. Furthermore, in the present study, the co-infiltration approach could result in variable expression levels for H chain and L chain in individual cells thereby resulting in free chains rather than assembled IgG. To address this, it will be possible to use a new generation of minimal binary vectors designed for use with CPMV-*HT* that allow both chains to be expressed together [43]. Alternatively, a DNA virus replicon system based on *Bean yellow dwarf virus* (BeYDV)[44] is also able to direct the transient expression both H and L chains from a single vector [33]. Nevertheless, considering that a one-step purification method was used here, the antigen binding abilities of the preparations are reasonable compared with ^CHO^2G12, which has undergone extra purification steps during its production according to clinically validated procedures.

Another property of RNA virus-directed replication that may compromise protein quality is the relatively low fidelity of RNA-dependent RNA polymerases (RdRp). RNA viruses are well known to accumulate mutations at a very high rate [45], which on one hand, allows them to quickly adapt to variations in environmental pressures [46], [47], but on the other hand, renders them susceptible to a very subtle decrease in RdRp fidelity, which can force a virus into error catastrophe [48], [49]. Consequently, for the use of virus vectors for the expression of therapeutics, there is concern over the accumulation of mutations during the multiple rounds of replication, and therefore the quality of the expressed proteins. The error rate per replication cycle of a number of RNA viruses have been reported to be in the range of approximately 1×10^−3^ to 1×10^−5^ mutations per base [48], [50], [51]. This suggests that a heterologous insert of 1 kb in a virus genome of 10 kb, will on average contain 0.01 to 1 mutations per replication cycle depending on the virus vector used. Cell to cell movement would then initiate a compounding replication cycle on any mutated genome. Furthermore, as there is no selection pressure against mutations in a heterologous insert, genetic drift is likely to occur. Given that some mutations will be synonymous and that the host cell possesses protein quality control mechanisms, it is currently very speculative to suggest that this will have a significant impact on the quality of a pharmaceutical protein produced by viral vectors. However, more detailed future studies will be required to demonstrate that replicating systems are robust and reliable enough to reproducibly deliver pharmaceuticals satisfying regulatory requirements. These concerns do not, of course, apply to systems such as CPMV-*HT*, which do not rely on RNA replication.

The results presented here demonstrate that it is possible to use transient expression systems based on CPMV to achieve the rapid, high level production of a high-quality therapeutic antibody. To our knowledge, this is the most detailed characterisation of transiently produced antibody from plants and the first to consider the effects of a virus vector on the quality of recombinant proteins from plants. The results show that in terms of HIV neutralisation, 2G12 produced by replicating and non-replicating CPMV expression systems are comparable to the CHO-produced antibody and 2G12 produced in transgenic plants. Furthermore, we believe that remaining quality issues can be resolved by further optimizing up- and downstream processing. The results might be taken as a first indication that the relatively high error rate of RdRp may be acceptable for the production of protein pharmaceuticals, although much more work is required to rigorously test this hypothesis. In conclusion, given the speed and ease with which large quantities of the recombinant antibody were produced, the transient expression systems detailed in this study are of great use for the research and development of pharmaceutical proteins whether ultimately produced in this manner or not.

## Materials and Methods

### Plasmids and agroinfiltration

The binary vector constructs used in this study have been previously described [26]. Briefly, sequences corresponding to the heavy and light chains of 2G12 were inserted into the cloning vector pM81-FSC1 using the appropriate restriction sites. This vector contains the CPMV RNA-2 sequence, from which the entire coding region may be replaced, flanked by a 35S promoter and *nos* terminator [52]. For expression with the wt RNA-2 leader (delRNA-2), *Pac*I/*Asc*I fragments containing the entire expression cassette were transferred into similarly digested pBINPLUS [53] to give pBD2G12H, pBD2G12HE and pBD2G12L (Figure 1). For expression with the CPMV-*HT* leader, *Dra*III/*Asc*I fragments containing the gene of interest, the 3′UTR, and the *nos* terminator were transferred into a similarly digested pBINPLUS construct carrying the *HT* mutation [26] to give pBD2G12H-*HT*, pBD2G12HE-*HT* and pBD2G12L-*HT* (Figure 1).

Expression plasmids were maintained in *Agrobacteria tumefaciens* strain LBA4404. Stationary phase liquid cultures grown in LB containing 50 µg/ml Kanamycin, 50 µg/ml Streptomycin, and 50 µg/ml Rifampicin were pelleted and resuspended to OD_600_ of 1.0 in MMA: 10 mM MES (2-[N-morpholino]ethanesulfonic acid; Sigma-Aldrich) pH 5.6, 10 mM MgCl_2_, 100 µM Acetosyringone (Sigma-Aldrich). Cultures harbouring binary plasmids for the expression of each antibody chain, RNA-1 and/or P19 were mixed at equal parts and pressure infiltrated into the underside of *N. benthamiana* leaves using a needleless syringe. Agroinfiltration was performed on all of the fully expanded leaves of 6 week-old plants grown in glasshouses maintained at 23–25°C with a 16 hour photoperiod.

### Antibody extraction and purification

Infiltrated leaves were homogenized in a Waring blender with 3 volumes of PBS (pH 6.0, 5 mM EDTA, 0.05% Triton X-100). After centrifugation (15 minutes at 8000 g) the supernatant was subjected to a pH shift to 8.5, resulting in formation of a precipitate. After a second centrifugation (30 minutes at 8000 g) the supernatant was passed through a paper filter and antibody was purified by Protein-A chromatography as previously described [54] with the exception that 100 mM glycine pH 3.6 with 100 mM fructose was used to elute from the Protein-A column. Eluates were immediately buffered with 1 M acetate buffer pH 4.75 and extensively dialysed against 10 mM acetate buffer pH 4.75 containing 1 mM ETDA. This buffer is similar to the buffer used for CHO-derived antibody (10 mM sodium acetate pH 4.5–5.0, 10% Maltose) and has been used for plant-derived 2G12 in previous studies (Ramessar et al., 2008). It has been found to be suitable for prolonged storage at 4°C without significant loss of antigen binding activity (data not shown). Concentration of the dialysed samples was determined by SPR and, if necessary, ultrafiltration (30 kDa MWCO) was used to concentrate the antibody.

### SDS-PAGE and Western blotting

2G12 preparations were separated by SDS-PAGE under reducing conditions [55] and either stained with Coomassie blue for direct visualisation or electroblotted onto nitrocellulose. Membranes were probed with either a goat anti-human IgG (Fc specific) conjugated to horseradish peroxidase (Sigma-Aldrich) or a goat anti-human IgG (Fab specific) conjugated to horseradish peroxidase (Sigma-Aldrich). Antigenic bands were visualized by electrochemiluminescence (ECL) captured on Hyperfilm (Amersham Biosciences).

### Surface Plasmon Resonance (SPR) spectroscopy

Antibody quantification, quality assessment, and antigen binding assays were carried out using a BIACORE 2000 instrument (Biacore, GE Healthcare) at 25°C using HBS-EP (10 mM HEPES, 150 mM NaCl, 3.4 mM EDTA, 0.05% Tween 20, pH 7.4) as the running buffer. Recombinant Protein-A (Sigma-Aldrich, 200 µg/ml in 10 mM sodium acetate, pH 4.2), Protein-L (Sigma-Aldrich, 62.5 µg/ml in 10 mM sodium acetate, pH 4.5) and gp120 HIV-1_BaL_ gp120, NIH AIDS Research and Reference Reagent Program, www.aidsreagent.org, 22.2 µg/ml in 10 mM sodium acetate pH 4.75) were immobilised to a CM5-rg sensorchip by EDC/NHS coupling and an activated/deactivated surface was used as a reference for blank subtraction. The contact times for activation and deactivation were increased to 10 min, and for coupling to 15 min to achieve high immobilization levels. Approximately 8.7 k Response Units (RU) of protein-A, 7.3 kRU protein-L, and 16.6 kRU of gp120 were immobilized, resulting in high binding capacity and high mass-transport limitation for all surfaces. Samples were diluted such that binding signals were in the linear range and dilution series were injected over all four cells simultaneously. Regeneration was achieved with 0.5 M citrate pH 3.0 for gp120 and 30 mM HCl for protein-A and protein-L, and surfaces were stable for several hundred cycles.

The response ratios were determined by linear regression of the endpoint signals recorded for several antibody concentrations after plotting the gp120 response against the protein-A and protein-L response, and the protein-L response against the protein-A response. The relative antigen-binding activity was derived by dividing the slopes by the corresponding slopes obtained for the ^CHO^2G12 reference (Polymun Scientific GmbH, Vienna, Austria). Data evaluation was performed using BIAevaluation v4.1 and Microcal Origin v8.0.

### Glycosylation analysis

Glycosylation analysis was carried out on the appropriate protein bands (either HC or full-size IgG) excised from a coomassie-stained SDS-PAGE gel. Destaining, carbamidomethylation, trypsin digest and extraction from gel pieces was performed as previously described [56]. Fractionation of the peptides by capillary reverse phase chromatography was also performed as described [56]. Comparison of MS data from the tryptic peptides was compared to tryptic digests of 2G12 performed *in silico* using the PeptideMass program (http://www.expasy.org/tools/peptide-mass.html). Tryptic glycopeptide datasets were generated by the addition of glycan mass increments to the masses of the two identified peptides.

### HIV neutralisation assay

HIV-1 neutralisation was assessed using a syncytium inhibition assay as previously described [14], [57]. Ten two-fold serial dilutions (start concentration: 100 µg/ml) of ^CHO^2G12, ^CPMV^2G12HL, ^CPMV^2G12HEL, ^HT^2G12HL, and ^HT^2G12HEL, and a non-neutralising control were pre-incubated with HIV-1 RF at 10^2^–10^3^ TCID_50_/ml for 1 hr at 37°C. CD4-positive human AA-2 cells were added at a density of 4×10^5^ cells/ml and incubated for a further 5 days. Experiments were performed with eight replicates per antibody dilution step. The presence of at least one syncytium per well was scored as positive for infection. The 50% inhibiting concentrations (IC_50_) were calculated according to the method of Reed and Muench [58] using the concentrations present during the antibody-virus pre-incubation step.

## Supporting Information

Figure S1 Effects of the presence of RNA-1 on the accumulation and glysosylation of 2G12 expressed by the delRNA-2 system. (A) Measurements of 2G12 accumulation made by SPR using a protein-A surface on crude plant extracts expressing secreted (HL) or ER retained (HEL) 2G12 in the presence or absence of RNA-1. Values represent the average of three replicates ± SE and is representative of 2 individual experiments. (B) Relative abundances of glycoforms of delRNA-2-produced 2G12 variants extracted 6 or 12 days after agro-infiltration and isolated from total soluble protein by 10% SDS-PAGE. H  =  heavy chain, L  =  light chain, HE  =  heavy chain with KDEL. *N*-glycan structure abbreviations are given according to http://www.proglycan.com.
(9.12 MB TIF)
