# Supplementary figures and images for: Rapid Transient Production in Plants by Replicating and Non-Replicating Vectors Yields High Quality Functional Anti-HIV Antibody
Source: PLoS One. 2010 Nov 12;5(11):e13976. doi: 10.1371/journal.pone.0013976 (PMC2980466; doi:10.1371/journal.pone.0013976)

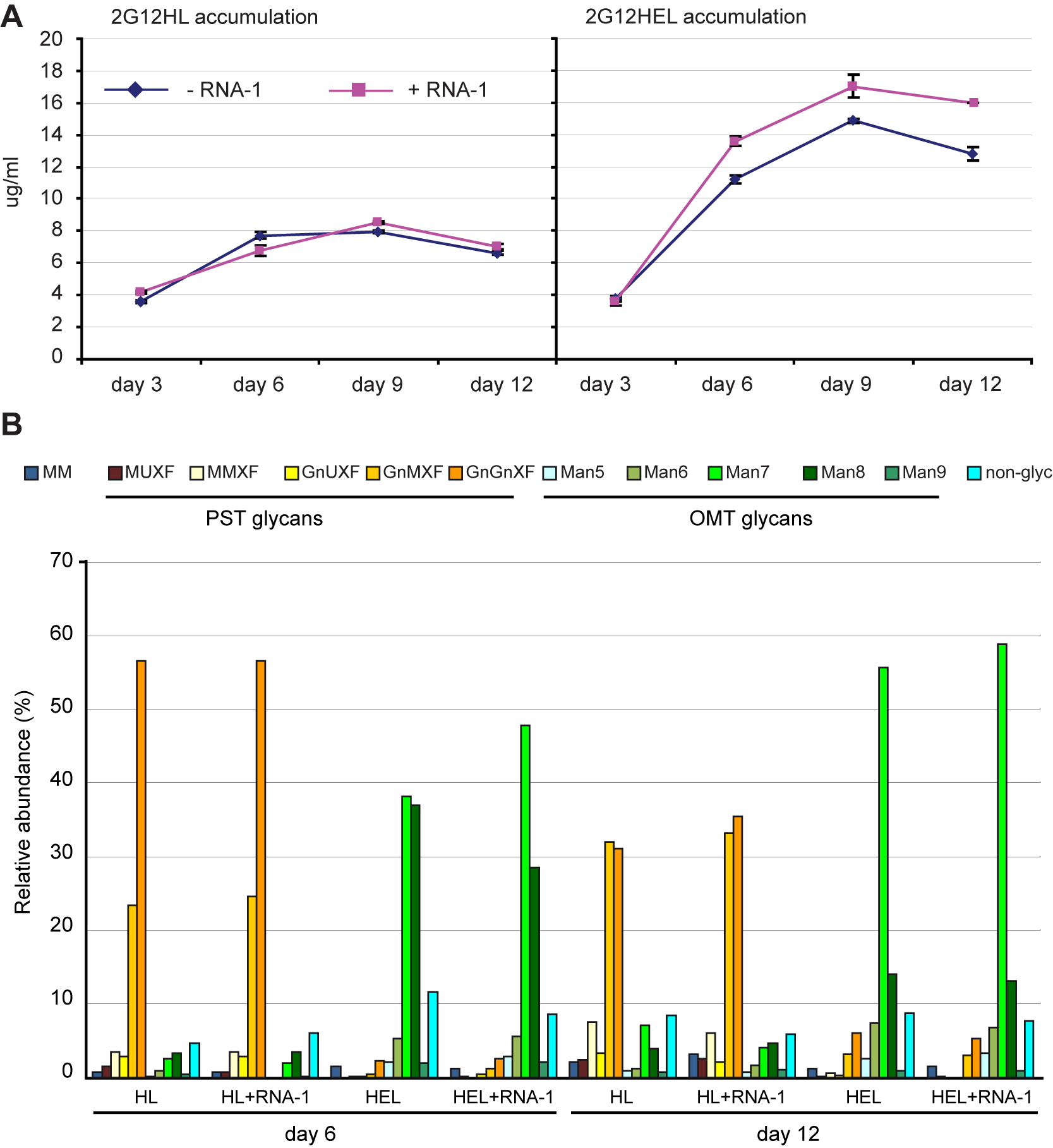

Supplement: Figure S1 — Effects of the presence of RNA-1 on the accumulation and glysosylation of 2G12 expressed by the delRNA-2 system. (A) Measurements of 2G12 accumulation made by SPR using a protein-A surface on crude plant extracts expressing secreted (HL) or ER retained (HEL) 2G12 in the presence or absence of RNA-1. Values represent the average of three replicates ± SE and is representative of 2 individual experiments. (B) Relative abundances of glycoforms of delRNA-2-produced 2G12 variants extracted 6 or 12 days after agro-infiltration and isolated from total soluble protein by 10% SDS-PAGE. H = heavy chain, L = light chain, HE = heavy chain with KDEL. N-glycan structure abbreviations are given according to http://www.proglycan.com. (9.12 MB TIF) [file pone.0013976.s001.tif]
